# Supplementary material for: Structure of the molecular bushing of the bacterial flagellar motor
Source: Nat Commun. 2021 Jul 22;12:4469. doi: 10.1038/s41467-021-24715-3 (PMC8298488; doi:10.1038/s41467-021-24715-3)
Supplement: Supplementary file 1 — Supplementary information [file 41467_2021_24715_MOESM1_ESM.pdf]

Supplementary Information

Structure of the molecular bushing of the bacterial flagellar motor

Tomoko Yamaguchi, Fumiaki Makino, Tomoko Miyata, Tohru Minamino, Takayuki Kato, Keiichi Namba

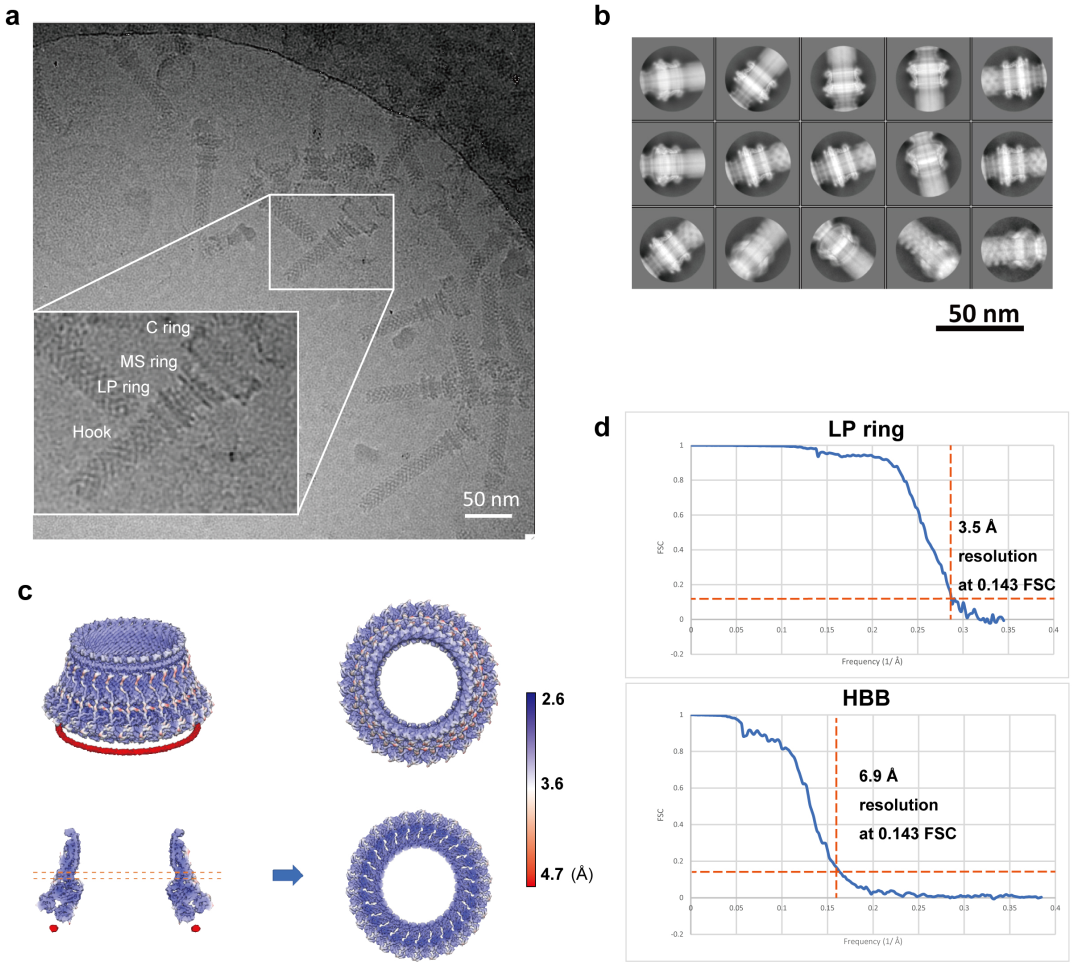

Supplementary Fig. 1 CryoEM single particle image analysis of the LP ring. **a**, Representative micrograph of the HBB complex of 12,759 micrographs we collected for image analysis. One of the HBB complex is magnified in the inset and the names of the structural parts are labeled. **b**, 2D class averages selected from 20 classes for 3D reconstruction of the LP ring with C26 symmetry. **c**, The local resolution of the final C26 LP ring density map colored from red (4.7 Å) to blue (2.6 Å). **d**, FSCs of the final density map of the LP ring reconstructed with C26 symmetry (upper) and the HBB complex with C1 symmetry (lower).

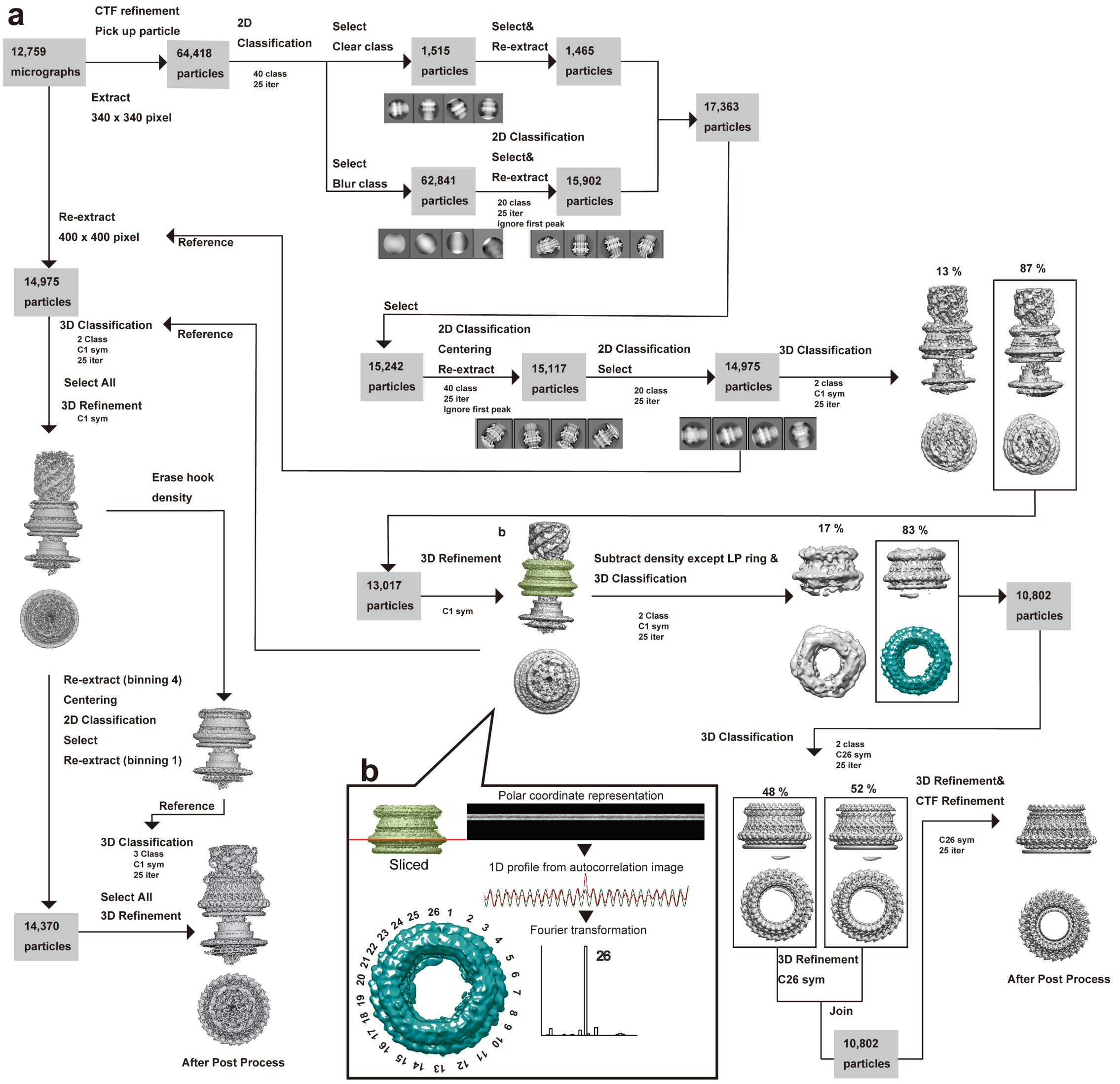

Supplementary Fig. 2 Work process of cryoEM 3D reconstruction of the HBB complex and LP ring. a, The work process of cryoEM single particle structural analysis of the HBB complex, in which 14,370 particles were used for final 3D classification and 3D refinement to produce a HBB density map at 6.9 Å resolution, and that of the LP ring alone, in which 10,802 particles were used to produce a density map at 3.5 Å resolution. **b**, The LP ring density colored light green was used to analyze its rotational symmetry. A horizontal slice of the LP ring density map at the red line was converted into the polar coordinate, and its auto-correlation was calculated and Fourier transformed to confirm the C26 symmetry indicated by visual inspection of the top view of the LP ring (green) extracted from the HBB complex reconstructed with C1 symmetry.

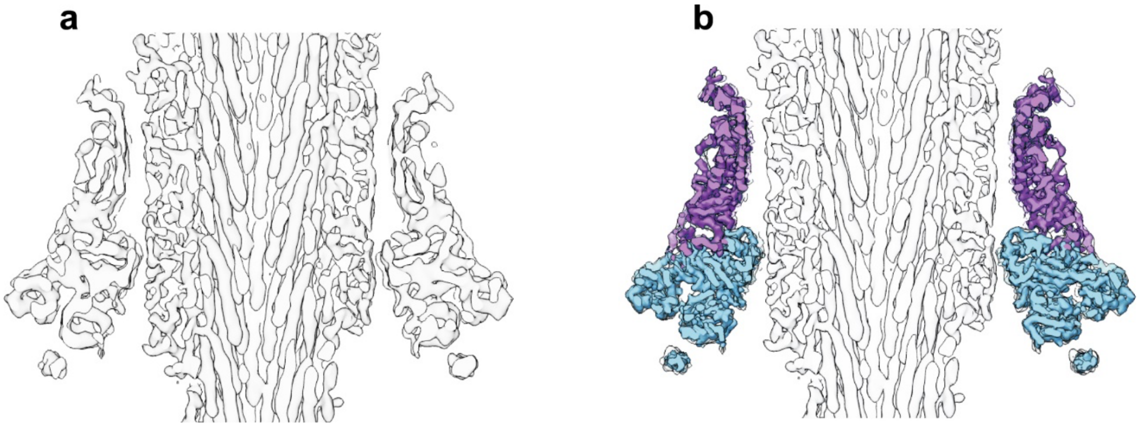

Supplementary Fig. 3 The LP ring position relative to the rod determined from the HBB complex density map. a, Vertical slice of the HBB complex density map (C1) including the LP ring and distal rod reconstructed at 6.9 Å resolution. **b**, The LP ring density map at 3.5 Å resolution (C26) is superimposed on the figure in **a**.

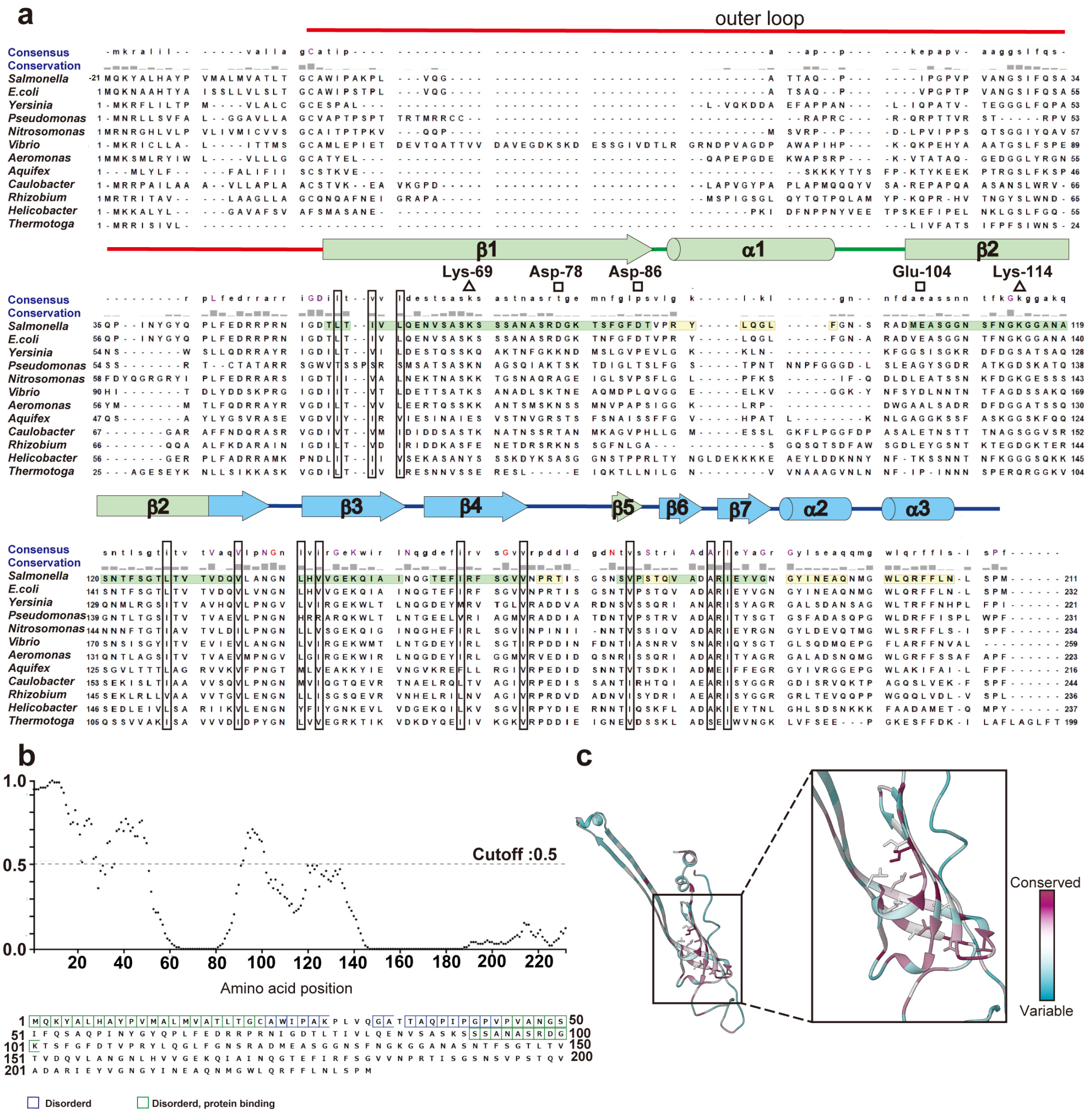

Supplementary Fig. 4 Multiple sequence alignment of FlgH. a, Multiple sequence alignment of FlgH carried out by Clustal Omega[1]. UniProt Accession numbers: *Salmonella* (*S. enterica* serovar Typhimurium), P0A1N8; *E. coli*, P0A6S0; *Yersinia* (*Y. pestis*), Q8ZHZ5; *Pseudomonas* (*P. putida*), Q52081; *Nitrosomonas* (*N. europaea*), Q82XG6; *Vibrio* (*V. parahaemolyticus*), Q9X9J5; *Aeromonas* (*A. hydrophila*), A0A142DZW5; *Aquifex* (*A. aeolicus*), O67609; *Caulobacter* (*C. vibrioides*), A0A0H3C9Y8; *Rhizobium* (*R. meliloti*), A0A222JND2; *Helicobacter* (*H. pylori*), E8QV65; *Thermotoga* (*T. maritima*), Q9X1M6. The five charged residues (Lys-69, Asp-78, Asp-86, Glu-104, Lys-114) and color-coded secondary structures of *Salmonella* FlgH in three domains (outer layer, red; inner layer, light green; middle layer, cyan) are shown above the sequence. **b**, Prediction of disordered regions by PSIPRED[2]. The disorder plot (upper) and predicted disordered regions (lower) agree with the outer layer chain of our atomic model. **c**, The sequence conservation of FlgH is colored from cyan to magenta (range of 0-100%), calculated and visualized by Chimera[3]. Side chains involved in intramolecular hydrophobic interactions are shown in stick representation and magnified (right), and these hydrophobic residues are also boxed in **a**.

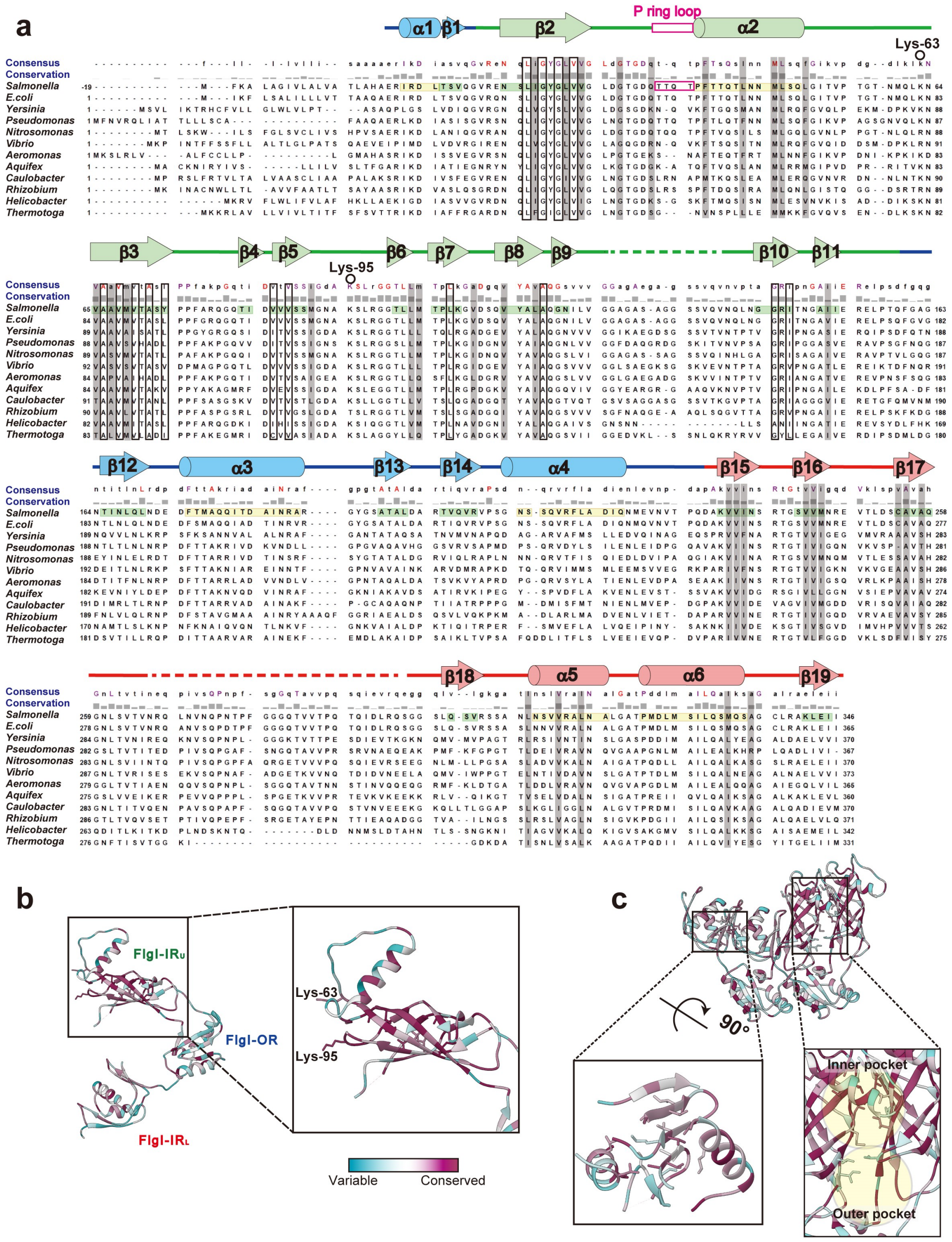

Supplementary Fig. 5 Multiple sequence alignment for FlgI. a, Multiple sequence alignment of FlgI carried out by Clustal Omega[1]. UniProt Accession numbers: *Salmonella* (*S. enterica* serovar Typhimurium), P15930; *E. coli*, P0A6S3; *Yersinia* (*Y. pestis*), Q8ZHZ4*; Pseudomonas* (*P. putida*), Q52082; *Nitrosomonas* (*N. europaea*), Q82XG5; *Vibrio* (*V. parahaemolyticus*), Q87JI2; *Aeromonas* (*A. hydrophila*), A0KM38; *Aquifex* (*A. aeolicus*), O67608; *Caulobacter* (*C. vibrioides*), P33979; *Rhizobium* (*R. meliloti*), Q52948; *Helicobacter* (*H. pylori*), O25028; *Thermotoga* (*T. maritima*), Q9X1M5. The two charged residues (Lys-63, Lys-95) and color-coded secondary structures of *Salmonella* FlgI with three domains (FlgI-OR, cyan; FlgI-IR$_U$, light green; FlgI-IR$_L$, red) are shown above the sequence. **b**, **c**, The sequence conservation of FlgI is colored from cyan to magenta (range of 0-100%), calculated and visualized by Chimera[3]. Side chains of Lys-63, Lys-95 and those involved in intra and intermolecular hydrophobic interactions are shown in stick representation and magnified in **b** and **c**, respectively, and these hydrophobic residues are also boxed and grey meshed in **a**, respectively.

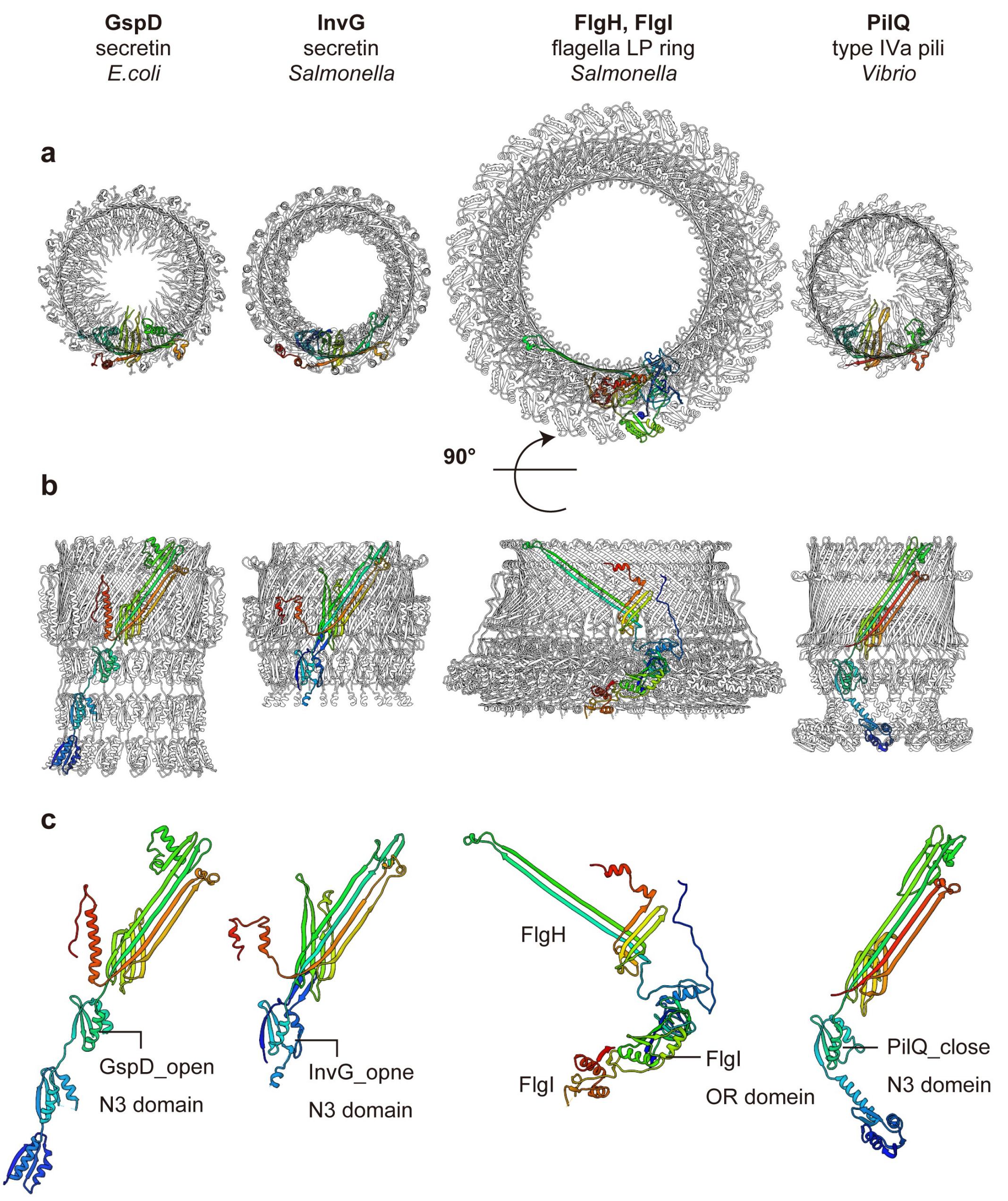

Supplementary Fig. 6 Structural comparison between the LP ring and rings of other secretion systems. a, **b**, Top and side views of GspD_open (residues 99-617, PDB ID: 5WQ7), InvG_open (residues 176–227, 252-557, PDB ID: 6DV6), the LP ring (PDB ID: 7CLR) and PilQ of type IVa pili (residues 160-571, PDB ID: 6W6M). **c,** Side view of each monomer colored rainbow.

a

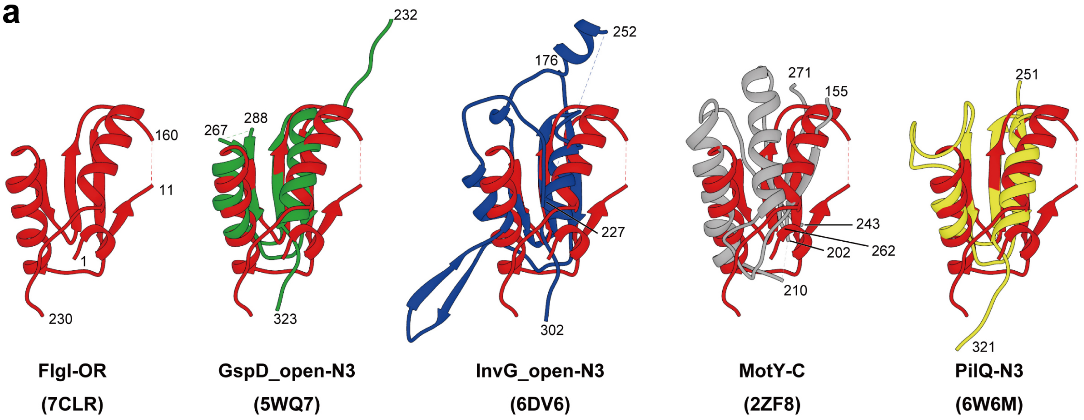

b

	GspD_open-N3	InvG_open-N3	MotY-C	PilQ-N3
[RMSD(Å)] root mean square deviation for all aligned regions	3.212	2.287	0.883	3.326
[SqID(%)] sequence identity for aligned regions	12.9	10.3	7.7	14.8

c

Color		PDB	Sequence
red	FlgI-OR	7CLR	1-11,160-230
green	GspD_open-N3	5WQ7	232-267, 288-323
blue	InvG_open-N3	6DV6	176-227, 252-302
gray	MotY-C	2ZF8	155-202, 210-243, 262-271
yellow	PilQ-N3	6W6M	251-321

d

```
Average secondary structure   c c t t s e e e e c b s s s s s s s b c h h h h h h h h h h h h h t t c c s c b g g g t b c c c c c c c t t c c c c
FlgI-OR                   1 : S F E D I A F T I L H Y E R Q G D Q L T K A S K K R L S Q I A D Y I R H N Q D - - - - - - - - - - - - - - - - - - - - - - : 193
GspD_open-N3            232 : - - - - - - - - - - - - - - - - - - - - - - - - - - - - - - - - - - - - - - E R I R D L T S V Q G F - - - - - - - - - - : 160
InvG_open-N3            176 : - - - - - - - - - - - - - - - - - - - - - - - - - - - - - - - - - - - - - - - - - - - - - - - - - - G - - - - - - - - - - : -
MotY-C                  155 : - - - - - - - - - - - - - - - - - - - - - - - - - - - - - - - - - - - - - - - - - - - - - - - - - - - L K S L D V E E : 176
PilQ-N3                 251 : - - - - - - - - - - - - - - - - - - - - - - - - - - - - - - - - - - - - - - - - - - - - - - - - - - - - - - - - - - - - : 239

Average secondary structure   c c c c e E E E e e c s s c c h s c h h h h c e e e e c s s c e e e e c h H H H H H h h h t s s h h h s h c c c c c c h
FlgI-OR                       : - - I D L V L V A T Y S A - - - S Q S L S E - - - - - - - - - - - - - - R R A E S L R D - - Y F Q S L G L - - - - - - - - : 232
GspD_open-N3                  : - G A G N T I N L Q L N D E D F T M - - A Q - - - - - - - - - - - - - - Q I T D A I N R - - - - - - - - A - - - - - - - - : 188
InvG_open-N3                  : - - R Q K I G V M R L N N - - - T F - - V G D R T Y N L R D Q K M V I P G I A T A I E R L L Q G E E Q P L G N I V S M S : 253
MotY-C                        : S E E G N T R V Y Y L K Y - - - A K - - A T - - - - - - - - - - - - - - N L V E V L T G - - - - - - - - V - - - - - - - - : 256
PilQ-N3                       : - - D L K S E I I K I N F - - - A K - - A S - - - - - - - - - - - - - - D I A A M I G G - - E G N V N M L - - - - - - - - : 280

Average secondary structure   h h h h t t s s c c c c s c e e e e E t t t t e E E E E c c t s h h c h h h h h h h h h h h h c c c c c c c c c c
FlgI-OR                       : - - - - - - - - - - P E D R I Q V Q G - - Y G R V V I S L G R - - T Q - - - - - - - - - - - - - - - - - - - - - -   : 271
GspD_open-N3                  : - - - - - - - - - - R G - Y G S A T A L D A R T V Q V R V P S G N S - S Q V R F L A D I Q N M E V N V T P Q - - -   : 230
InvG_open-N3                  : L Q E A L K Q N A A A G N I K I V A Y P D T N S L L V K G T A - - E - Q V H F I E M L V K A L D V A K - - - R - -   : 302
MotY-C                        : - - - - - - - - - - S E - V A I T A D E Q T N S L V I T A D Q - - S - V Q E K L A T V I A R L D I R R - - - A - -   : 323
PilQ-N3                       : - - - - - - - - - - S E R G S I S I D E R T N S L L I R E L P - - D - N I A V I R E I I E S L D I P V - - - K Q V   : 321
```

Supplementary Fig. 7 Structural comparison between FlgI-OR and related domains of other secretion systems. a, Superposition of FlgI-OR (residues 1–11, 160–230: red) on to GspD_open-N3 (residues 232–267, 288-323: green), InvG_open-N3 (residues 176–227, 252-302: blue), MotY-C (PDB ID: 2ZF8, residue 155-202, 210-243, 262-271: gray) and PilQ-N3 (residues 251-321: yellow). **b**, Alignment score between FlgI-OR and other four domains. **c**, Color, Name, PDB ID, and sequence region of compared domains. **d**, Sequence alignment between FlgI-OR and other four domains. All the alignment and numbers in the tables were calculated by MATRAS[4].

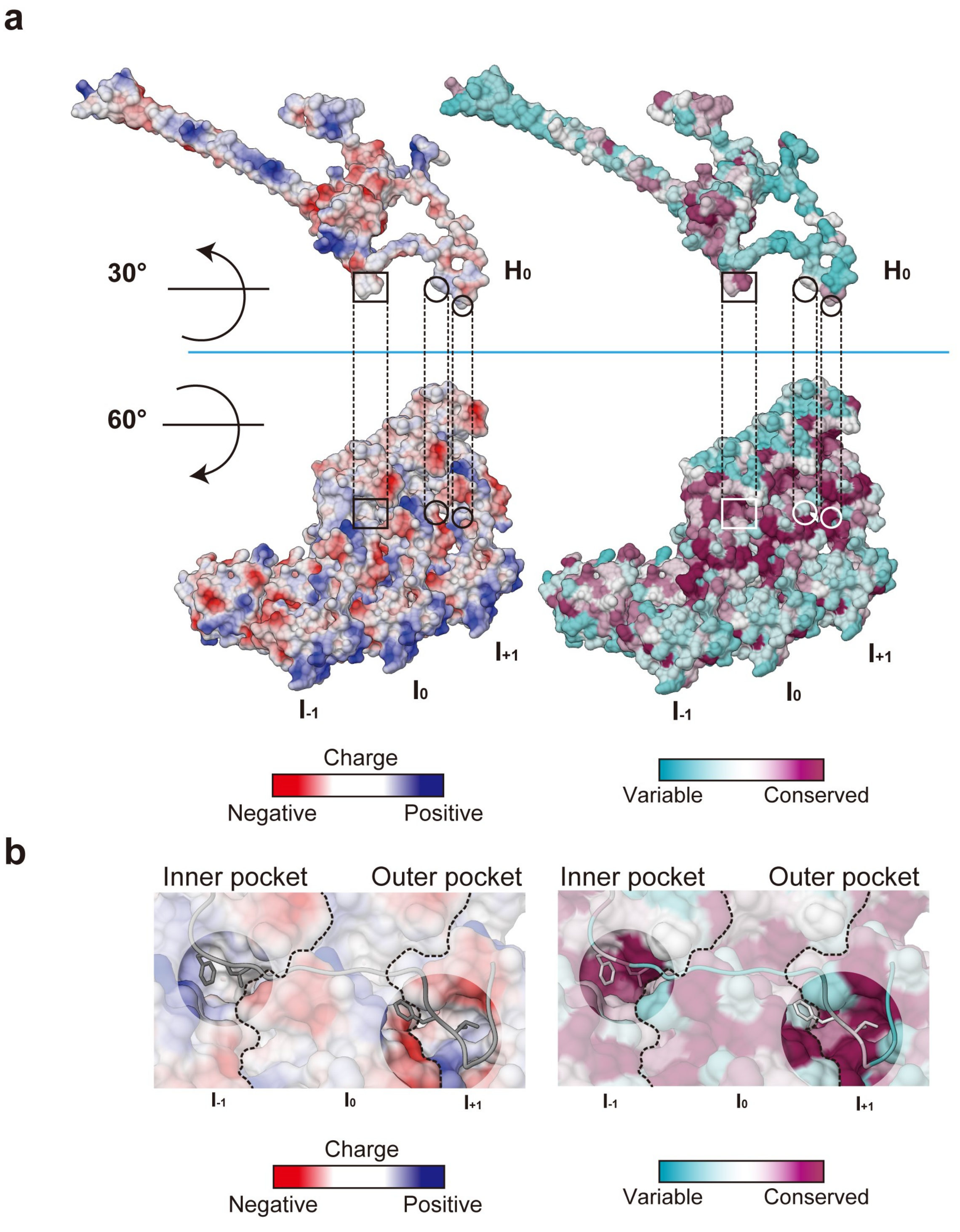

Supplementary Fig. 8 Electrostatic surface potential and sequence conservation of FlgH and FlgI. a, Interactions between one FlgH and three FlgI (I_0, $I_{\pm1}$) molecules. The interactions in the inner pocket (square) and outer pocket (circle) are both hydrophobic (left), and the residues forming them are mostly highly conserved (right). **b**, Enlarged views of the interactions in the inner pocket and outer pocket. Three FlgI (I_0, $I_{\pm1}$) molecules are displayed as solid surface and FlgH as Cα ribbon. The hydrophobic side chains of FlgH are displayed in stick and are color-coded as gray in the electrostatic surface potential (left) and cyan to magenta in the conservation (right). All figures are made by Chimera[3]

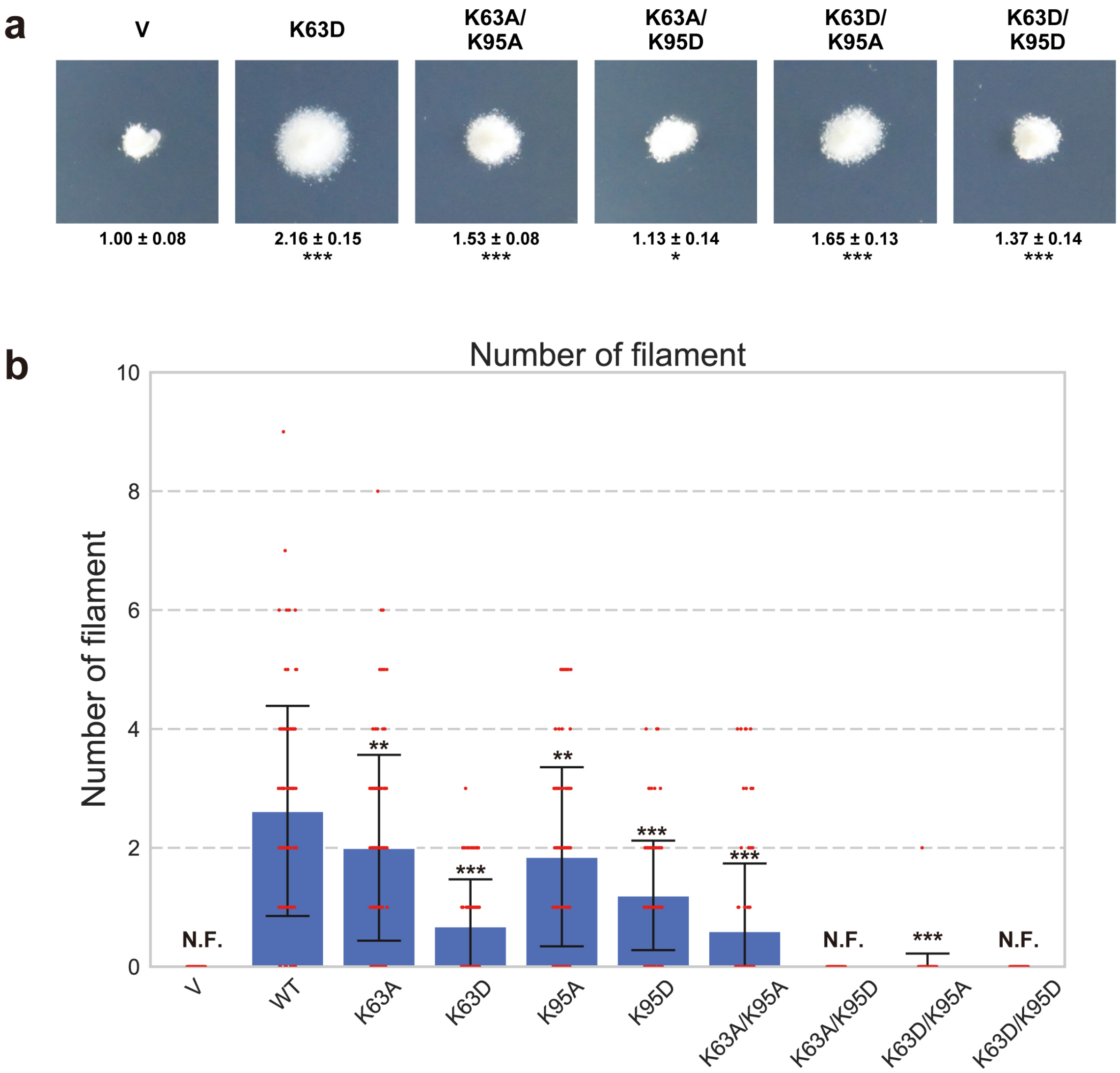

Supplementary Fig. 9 Motility and filament number assay of *flgI* mutants. a, Motility assay in 0.35% soft agar plate of the *flgI(K63D)*, *flgI(K63A/K95A)*, *flgI(K63A/K95D)*, *flgI(K63D/K95A)* and *flgI(K63D/K95D)* mutants. The diameter of motility ring was measured for eight colonies of each strain after incubating 24 hours at 30°C and compared with the vector control, V. The average diameter of the motility ring of the vector strain V was set to 1.0, and then the diameters of the motility rings of mutant cells were compared with that of V (mean ± SD, $n = 8$). Comparisons between datasets were performed using a two-tailed Student's *t*-test. P value of < 0.05 was considered to be statistically significant difference. *, $P < 0.05$; ***, $P < 0.001$. **b,** Number of flagellar filaments per cell for the *flgI(K63A), flgI(K63D)*, *flgI(K95A), flgI(K95D), flgI(K63A/K95A)*, *flgI(K63A/K95D)*, *flgI(K63D/K95A)* and *flgI(K63D/K95D)* mutant strains. The letters V and WT refer to the empty expression vector (pET22b) and wild type *flgI* expression vector pTY03 (Supplementary Table 1) for the negative and positive controls, respectively. Overnight cultured cells were stained by 0.2% phosphotungstic acid and observed by electron microscopy. The number of filaments were counted and compared with WT (mean ± SD, $n = 100$). Comparisons between datasets were performed using a two-tailed Student's *t*-test. P value of < 0.05 was considered to be statistically significant difference. **, $P < 0.01$; ***, $P < 0.001$. N.F., no flagellum.

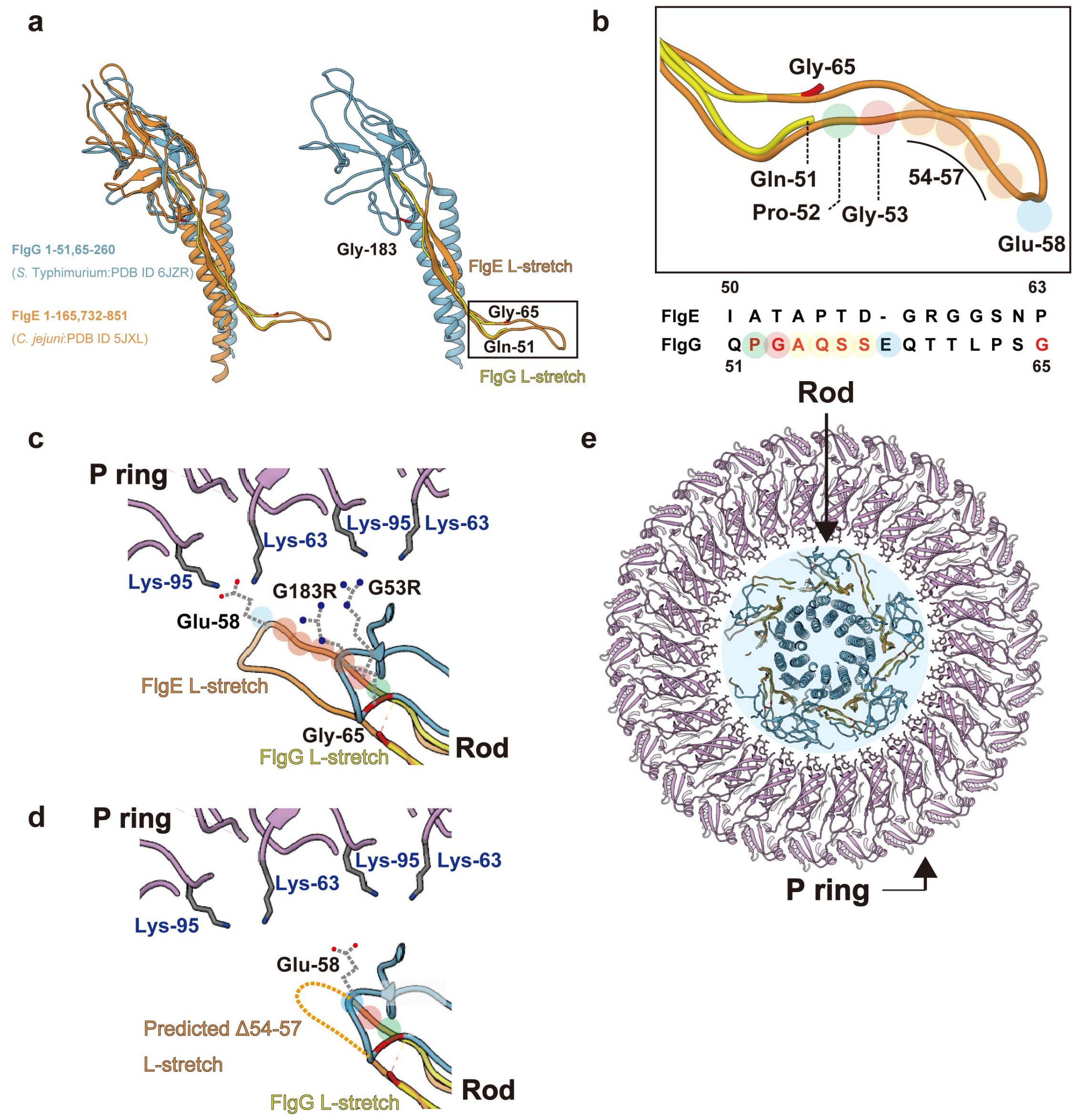

Supplementary Fig. 10 Interactions between FlgG in the rod and FlgI in the P ring. a, Structural comparison between FlgG of *S. enterica* (PDB ID: 6JZR) and FlgE (residues 1-165 and 732-851) of *C. jejuni* (PDB ID: 5JXL) as a full-length structural representative of FlgG. The FlgE L-stretch (residues 31–80) is superimposed to FlgG L-stretch (residues 32–82) with RMSD 1.16 Å. **b**, The position of the missing residues of the FlgG model (residues 52–58) in the L-stretch, with residues predicted to interact with FlgI in the P ring displayed by colored circles. **c**, Possible interactions of Lys-63 and Lys-95 of FlgI in the P ring and Glu-58, Arg-183 and Arg-53 of FlgG in the rod. Glu-58 is expected to interact with Lys-63 and Lys-95 and helps P ring assembly. The G183R and G53R mutation of FlgG is expected to disturb P ring assembly by repulsive force. **d**, Predicted change of the Glu-58 position in the Δ54-57 mutant of FlgG, making the distance between Glu-58 of FlgG and Lys-63 and Lys-95 of FlgI too far for interaction. **e**, Horizontal slice of the rod and P ring at their closest position, with side chains of FlgI in the P ring loop displayed in stick representation. There is still a small gap between the rod and P ring to accommodate water molecules.

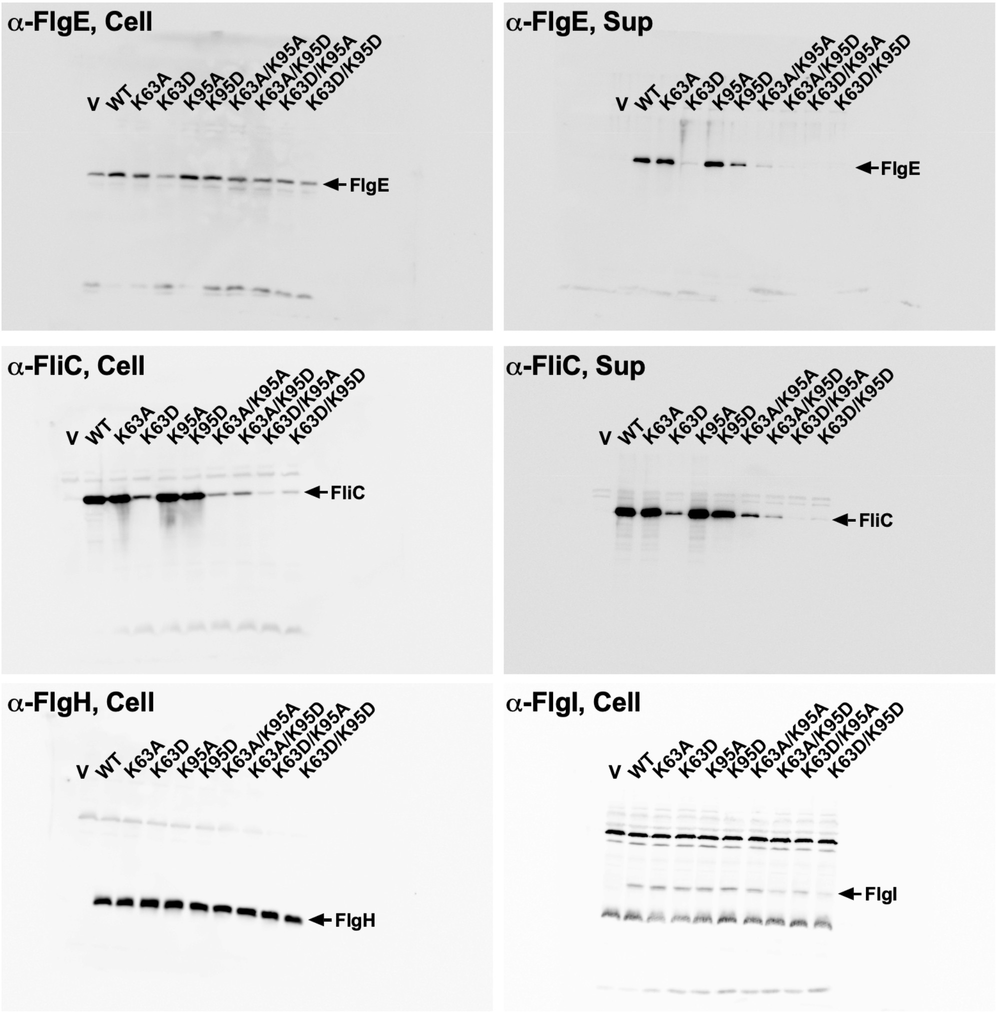

Supplementary Fig. 11 Original immunoblots of data shown in Fig. 5b. The antibodies used in the immunoblots are shown in top left of each panel. Labels "Cell" and "Sup" indicate the fractions of proteins in the cell and culture supernatant, respectively. The proteins identified by immunoblotting are indicated by arrows with labels of protein names. The letters V and WT refer to the empty expression vector (pET22b) and wiled type *flgI* expression vector (pTY03) (Supplementary Table 1).

Supplementary Table 1 Strains and plasmids used in this study

**Strains and plasmid**	**Relevant characteristics**	**Source or reference**
***Salmonella* strains**		
HK1002	Not forming the filament<br>Δ*flgK*, Δ*cliP::*Cm^r	Ref.[5]
SJW203	LP ring deletion mutant<br>Δ*flgH-flgI*	Shigeru Yamaguchi<br>& Ref.[6]
**Plasmids**		
pET22b	Expression vector	Novagen
pTY03	pET22b/FlgH+FlgI	This study
pTY03(K63A)	pET22b/FlgH+FlgI(K63A)	This study
pTY03(K63D)	pET22b/FlgH+FlgI(K63D)	This study
pTY03(K95A)	pET22b/FlgH+FlgI(K95A)	This study
pTY03(K95D)	pET22b/FlgH+FlgI(K95D)	This study
pTY03(K63A/K95A)	pET22b/FlgH+FlgI(K63A/K95A)	This study
pTY03(K63A/K95D)	pET22b/FlgH+FlgI(K63A/K95D)	This study
pTY03(K63D/K95A)	pET22b/FlgH+FlgI(K63D/K95A)	This study
pTY03(K63D/K95D)	pET22b/FlgH+FlgI(K63D/K95D)	This study

Supplementary Table 2 Primers used in this study

Mutated residue	Primer	Sequence (5'-3')
	FlgHI_NdeI_Fw-2	CATATGCAAAAATACGCGCTTCACGC
	FlgHI_BamHI_Rv-3	CTCGGATCCTCAGATGATTTCCAGTTTGGCGCG
K63A	FlgI_K63A_Fw	CAGTTGGCAAACGTGGCGGCGGTGATGGTGACG
	FlgI_K63A_Rv	CACGTTTGCCAACTGCATATTGGTGCCGGTGGG
K63D	FlgI_K63D_Fw	CAGTTGGATAACGTGGCGGCGGTGATGGTGACG
	FlgI_K63D_Rv	CACGTTATCCAACTGCATATTGGTGCCGGTGGG
K95A	FlgI_K95A_Fw	AACGCTGCAAGTCTGCGTGGCGGGACGTTATTA
	FlgI_K95A_Rv	CAGACTTGCAGCGTTCCCCATTGAGGAAACAAC
K95D	FlgI_K95D_Fw	AACGCTGATAGTCTGCGTGGCGGGACGTTATTA
	FlgI_K95D_Rv	CAGACTATCAGCGTTCCCCATTGAGGAAACAACGAC
K63A/ K95A	FlgI_K63A_Fw	CAGTTGGCAAACGTGGCGGCGGTGATGGTGACG
	FlgI_K63A_Rv	CACGTTTGCCAACTGCATATTGGTGCCGGTGGG
	FlgI_K95A_Fw	AACGCTGCAAGTCTGCGTGGCGGGACGTTATTA
	FlgI_K95A_Rv	CAGACTTGCAGCGTTCCCCATTGAGGAAACAAC
K63A/K95D	FlgI_K63A_Fw	CAGTTGGCAAACGTGGCGGCGGTGATGGTGACG
	FlgI_K63A_Rv	CACGTTTGCCAACTGCATATTGGTGCCGGTGGG
	FlgI_K95D_Fw	AACGCTGATAGTCTGCGTGGCGGGACGTTATTA
	FlgI_K95D_Rv	CAGACTATCAGCGTTCCCCATTGAGGAAACAACGAC
K63D/K95A	FlgI_K63D_Fw	CAGTTGGATAACGTGGCGGCGGTGATGGTGACG
	FlgI_K63D_Rv	CACGTTATCCAACTGCATATTGGTGCCGGTGGG
	FlgI_K95A_Fw	AACGCTGCAAGTCTGCGTGGCGGGACGTTATTA
	FlgI_K95A_Rv	CAGACTTGCAGCGTTCCCCATTGAGGAAACAAC
K63D/K95D	FlgI_K63D_Fw	CAGTTGGATAACGTGGCGGCGGTGATGGTGACG
	FlgI_K63D_Rv	CACGTTATCCAACTGCATATTGGTGCCGGTGGG
	FlgI_K95D_Fw	AACGCTGATAGTCTGCGTGGCGGGACGTTATTA
	FlgI_K95D_Rv	CAGACTATCAGCGTTCCCCATTGAGGAAACAACGAC

Supplementary Table 3 CryoEM data collection, refinement and validation statistics

	HBB (EMDB-30409)	LP ring (EMDB-30398) (PDB 7CLR)
**Data collection and processing**		
Magnification	40,000	40,000
Voltage (kV)	200	200
Total exposure time (sec)	10	10
Pixel size (Å)	1.45	1.45
Total electron exposure (e^{-}/Å^2)	45	45
Number of frames	50	50
Dose rate (e^{-}/Å^2/frame)	0.9	0.9
Number of micrographs	12,759	12,759
Defocus range (μm)	0.2-2.0	0.2-2.0
Symmetry imposed	C1	C26
Initial particle images (no.)	64,418	64,418
Final particle images (no.)	14,370	10,802
Map resolution (Å)	6.9	3.5
FSC threshold	0.143	0.143
Map resolution range (Å)	4.14-15.9	2.42-4.05
**Refinement**		
Map sharpening *B* factor (Å^2)	-88.05	-38.08
Model composition		
Non-hydrogen atoms		12,480
Protein residues		12,480
Ligands		0
R.m.s. deviations		
Bond lengths (Å)		0.50
Bond angles (°)		0.69
Validation		
MolProbity score		2.32
Clashscore		6.4
Poor rotamers (%)		5.5
Ramachandran plot		
Favored (%)		93.9
Allowed (%)		6.15
Disallowed (%)		0

Supplementary Table 4 The exact *P* values of two-tailed Student's *t*-test shown in Fig. 5 and Supplementally Fig. 9

**Swarm assay shown in Fig. 5a (*n* =5)**		
**Sample 1**	**Sample 2**	***P* value**
WT	K63A	3.8×10^{-6}
WT	K63D	2.0×10^{-9}
WT	K95A	4.6×10^{-4}
WT	K95D	3.1×10^{-7}
V	K63D	1.3×10^{-4}
V	K63A/K95A	2.6×10^{-2}
V	K63A/K95D	5.5×10^{-2}
V	K63D/K95A	1.4×10^{-1}
V	K63D/K95D	5.3×10^{-1}
**Secretion assay of FlgE shown in Fig. 5c (*n*=5)**		
**Sample 1**	**Sample 2**	***P* value**
WT	K63A	5.6×10^{-1}
WT	K63D	2.4×10^{-5}
WT	K95A	4.3×10^{-1}
WT	K95D	4.6×10^{-3}
WT	K63A/K95A	7.6×10^{-6}
WT	K63A/K95D	5.3×10^{-6}
WT	K63D/K95A	4.7×10^{-6}
WT	K63D/K95AD	4.4×10^{-6}
**Secretion assay of FliC shown in Fig. 5c (*n*=5)**		
**Sample 1**	**Sample 2**	***P* value**
WT	K63A	9.7×10^{-1}
WT	K63D	2.8×10^{-4}
WT	K95A	5.1×10^{-1}
WT	K95D	2.1×10^{-1}
WT	K63A/K95A	2.4×10^{-4}
WT	K63A/K95D	2.7×10^{-5}
WT	K63D/K95A	3.6×10^{-5}
WT	K63D/K95AD	3.6×10^{-5}

**Swarm assay shown in Fig. 9a (*n* =8)**		
**Sample 1**	**Sample 2**	***P* value**
V	K63D	2.3×10^{-11}
V	K63A/K95	2.7×10^{-9}
V	K63A/K95D	3.7×10^{-2}
V	K63D/K95A	1.1×10^{-8}
V	K63D/K95D	1.3×10^{-5}
**Flagellar number per cell shown in Fig. 9b (*n* =100)**		
**Sample 1**	**Sample 2**	***P* value**
WT	K63A	9.3×10^{-3}
WT	K63D	2.3×10^{-19}
WT	K95A	1.1×10^{-3}
WT	K95D	1.9×10^{-11}
WT	K63A/K95A	3.5×10^{-18}
WT	K63D/K95A	2.8×10^{-33}

Supplementary References

1. Sievers, F. *et al.* Fast, scalable generation of high-quality protein multiple sequence alignments using Clustal Omega. *Mol. Syst. Biol.* **7**, 539 (2011).
2. Jones, D. T. Protein secondary structure prediction based on position-specific scoring matrices. *J. Mol. Biol.* **292**, 195–202 (1999).
3. Pettersen, E. F. *et al.* UCSF Chimera - A visualization system for exploratory research and analysis. *J. Comput. Chem.* **25**, 1605–1612 (2004).
4. Kawabata, T. MATRAS: A program for protein 3D structure comparison. *Nucleic Acids Res.* **31**, 3367–3369 (2003).
5. Kawamoto, A. *et al.* Common and distinct structural features of *Salmonella* injectisome and flagellar basal body. *Sci. Rep.* **3**, 3369 (2013).
6. Karlinsey, J. E., Pease, A. J., Winkler, M. E., Bailey, J. L. & Hughes, K. T. The *flk* gene of *Salmonella typhimurium* couples flagellar P-and L-ring assembly to flagellar morphogenesis. *J. Bacteriol.* **179**, 2389–2400 (1997).
