# Supplementary material for: Structure of the molecular bushing of the bacterial flagellar motor
Source: Nat Commun. 2021 Jul 22;12:4469. doi: 10.1038/s41467-021-24715-3 (PMC8298488; doi:10.1038/s41467-021-24715-3)
Supplement: Supplementary file 3 — Description of Additional Supplementary Files [file 41467_2021_24715_MOESM3_ESM.pdf]

Description of Additional Supplementary Files

File Name: Supplementary Movie 1
Description: **Structure of Salmonella flagellar LP ring and its component proteins FliH and FliI.** The 3D density map of the LP ring obtained by cryoEM image analysis and atomic models of its component proteins FliH and FliI in Cα ribbon representation colored in rainbow according to their sequences.
