# Supplementary material for: Structure of the molecular bushing of the bacterial flagellar motor
Source: Nat Commun. 2021 Jul 22;12:4469. doi: 10.1038/s41467-021-24715-3 (PMC8298488; doi:10.1038/s41467-021-24715-3)
Supplement: Supplementary file 5 — Reporting Summary [file 41467_2021_24715_MOESM5_ESM.pdf]

nature research

Corresponding author(s): Keiichi Namba

Last updated by author(s): Jun 21, 2021

Reporting Summary

Nature Research wishes to improve the reproducibility of the work that we publish. This form provides structure for consistency and transparency in reporting. For further information on Nature Research policies, see Authors & Referees and the Editorial Policy Checklist .

Statistics

For all statistical analyses, confirm that the following items are present in the figure legend, table legend, main text, or Methods section.

n/a	Confirmed	
☐	☒	The exact sample size (*n*) for each experimental group/condition, given as a discrete number and unit of measurement
☐	☒	A statement on whether measurements were taken from distinct samples or whether the same sample was measured repeatedly
☐	☒	The statistical test(s) used AND whether they are one- or two-sided<br>*Only common tests should be described solely by name; describe more complex techniques in the Methods section.*
☐	☒	A description of all covariates tested
☒	☐	A description of any assumptions or corrections, such as tests of normality and adjustment for multiple comparisons
☐	☒	A full description of the statistical parameters including central tendency (e.g. means) or other basic estimates (e.g. regression coefficient) AND variation (e.g. standard deviation) or associated estimates of uncertainty (e.g. confidence intervals)
☐	☒	For null hypothesis testing, the test statistic (e.g. *F*, *t*, *r*) with confidence intervals, effect sizes, degrees of freedom and *P* value noted<br>*Give P values as exact values whenever suitable.*
☒	☐	For Bayesian analysis, information on the choice of priors and Markov chain Monte Carlo settings
☒	☐	For hierarchical and complex designs, identification of the appropriate level for tests and full reporting of outcomes
☒	☐	Estimates of effect sizes (e.g. Cohen's *d*, Pearson's *r*), indicating how they were calculated

Our web collection on statistics for biologists contains articles on many of the points above.

Software and code

Policy information about availability of computer code

Data collection	All images were collected by JADAS v 4.0.0.3004, which is an automated data accusation program produced by JEOL.
Data analysis	The cryoEM movies were taken automatically by JADAS v4.0.0.3004 (JEOL, Japan). CTF estimation was carried out by Gctf v1.06. The particle images were picked up using an in-house particle picking program applying a deep learning method based on YOLO neural network. REION v3.0-β2 was used for motion correction of the movie, 2D and 3D classification, 3D refinement, CTF refinement and estimation of local resolution. Atomic model building was performed by using COOT v0.8.9.1 and UCSF Chimera v1.13.1. PHENIX v1.13.2998 was used for auto sharpening of the cryoEM map and real-space refinement. PSIPRED (http://bioinf.cs.ucl.ac.uk/psipred/) was used for secondary structure prediction. The APBS and PDB2PQR plugged in the PyMol v2.3.2 was used for calculating the surface potential . Clustal Omega v1.2.4 was used for multi sequence alignment. MATRAS (http://strcomp.protein.osaka-u.ac.jp/matras/) was used for 3D structure comparison of the atomic models. A diameter of the motility ring of each Salmonella strain was measured by ImageJ v 1.52 (National Institutes of Health). Band intensities on blots were measured using an image analysis software, CS Analyzer 4 (ATTO, Tokyo, Japan). Statistical analyses were done using KaleidaGraph (HULINKS) or Excel 2016 (Microsoft). 3D models were visualized by UCSF Chimera v1.13.1. All figures were created by using Photoshop 2020 and Illustrator 2021 (Adobe).

For manuscripts utilizing custom algorithms or software that are central to the research but not yet described in published literature, software must be made available to editors/reviewers. We strongly encourage code deposition in a community repository (e.g. GitHub). See the Nature Research guidelines for submitting code & software for further information.

Data

Policy information about availability of data

All manuscripts must include a data availability statement. This statement should provide the following information, where applicable:

- Accession codes, unique identifiers, or web links for publicly available datasets
- A list of figures that have associated raw data
- A description of any restrictions on data availability

The atomic model and density map of the LP ring have been deposited in PDB database under accession code 7CLR [https://doi.org/10.2210/pdb7CLR/pdb] and EMDB data base under accession code EMD-30398 [https://www.ebi.ac.uk/pdbe/entry/emdb/EMD-30398], respectively. The HBB density map has been deposited in EMDB database under accession code EMD-30409 (HBB) [https://www.ebi.ac.uk/pdbe/entry/emdb/EMD-30409]. All the other data supporting the findings of this study are available within the article and its Supplementary Information/Source Data file. A reporting summary for this article is available as a Supplementary Information file. Source data are provided with this paper.

Field-specific reporting

Please select the one below that is the best fit for your research. If you are not sure, read the appropriate sections before making your selection.

- [x] Life sciences
- [ ] Behavioural & social sciences
- [ ] Ecological, evolutionary & environmental sciences

For a reference copy of the document with all sections, see nature.com/documents/nr-reporting-summary-flat.pdf

Life sciences study design

All studies must disclose on these points even when the disclosure is negative.

Sample size	We disclosed the sample size in this article and its Supplementary Information files. Totally 12,759 of movie files were recorded in one beam time, 64,418 particle images that were recognized as the LP ring by an in-house python program (YOLOPick.py) were automatically picked up and used for further analysis. Among these, 14,370 and 10,802 particle images of the HBB and LP ring, respectively, were used for final reconstruction because they were classified into higher resolution datasets after 3D classification by RELION. No statistical methods were used to predetermine the sample size.
Data exclusions	By 2D and 3D classification and selection, 50,048 of the HBB particle images and 53,616 of the LP ring particle images were excluded from the initial 64,418 particle images.
Replication	All attempts at replication were successful for structural analysis .We carried out at least five independent measurements to confirm the reproducibility of motility and biochemical assay.
Randomization	We allocated Salmonella strains into experimental groups based on their genetic background. In each group, the samples were selected randomly. Randomization is not relevant to structural analysis, as the protein is not required to be allocated into experimental groups.
Blinding	Blinding is not relevant to this study, as the protein is not required to be allocated into experimental groups in structural analysis.

Reporting for specific materials, systems and methods

We require information from authors about some types of materials, experimental systems and methods used in many studies. Here, indicate whether each material, system or method listed is relevant to your study. If you are not sure if a list item applies to your research, read the appropriate section before selecting a response.

Materials & experimental systems

n/a	Involved in the study	
[ ]	[x]	Antibodies
[x]	[ ]	Eukaryotic cell lines
[x]	[ ]	Palaeontology
[x]	[ ]	Animals and other organisms
[x]	[ ]	Human research participants
[x]	[ ]	Clinical data

Methods

n/a	Involved in the study	
[x]	[ ]	ChIP-seq
[x]	[ ]	Flow cytometry
[x]	[ ]	MRI-based neuroimaging

Antibodies

Antibodies used	We used polyclonal anti-FlgH, anti- FlgI, anti- FlgE or anti- FliC antibody as the primary antibody for immunoblotting. We sent purified FlgH, FlgI, FlgE and FliC proteins to Medical & Biological Laboratories Co., LTD. (https://www.mblbio.com/e/) to prepare the antibodies. We also used anti-Rabbit IgG, HRP-Linked Whole Ab Donkey (GE Healthcare, Cat#SE250-10A-.75) as the secondary antibody.

Validation

Primary antibodies were raised against Salmonella flagellar proteins. These antibodies have already been published in our previous papers.
